# Recurrent Severe Subclinical Mastitis and the Risk of HIV Transmission Through Breastfeeding

**DOI:** 10.3389/fimmu.2022.822076

**Published:** 2022-03-04

**Authors:** David Gatsinzi Rutagwera, Jean-Pierre Molès, Chipepo Kankasa, Mwiya Mwiya, Edouard Tuaillon, Marianne Peries, Nicolas Nagot, Philippe Van de Perre, Thorkild Tylleskär

**Affiliations:** ^1^Centre for International Health, University of Bergen, Bergen, Norway; ^2^Children’s Hospital, University Teaching Hospitals, School of Medicine, University of Zambia, Lusaka, Zambia; ^3^Pathogenesis and Control of Chronic and Emerging Infections, Université de Montpellier, INSERM, Université des Antilles, Etablissement français du Sang, Montpellier, France

**Keywords:** breastfeeding, HIV-1 transmission, HIV-1 shedding, inflammation, cytokine

## Abstract

Subclinical mastitis (SCM) is an important risk factor of postnatal HIV-1 transmission that is still poorly understood. A longitudinal sub-study of the ANRS12174 trial including 270 breastfeeding mothers in Lusaka, Zambia measured sodium (Na^+^) and potassium (K^+^) in archived paired breast milk samples collected at week 14, 26 and 38 postpartum to determine cumulative incidence of SCM and the effects of recurrent severe SCM on HIV-1 shedding in breast milk. A nested retrospective cohort study including 112 mothers was also done to determine longitudinal effects of SCM on four pro-inflammatory cytokines; IL6, IL8, IP10 and RANTES. The cumulative incidence for any SCM (Na**^+^**/K**^+^** ratio > 0.6) and severe SCM (Na**^+^**/K**^+^** ratio > 1) were 58.6% (95%CI: 52.7 – 64.5) and 27.8% (95%CI: 22.5 – 33.1), respectively. In majority of affected mothers (51.4%) severe SCM was recurrent. Both breasts were involved in 11.1%, 33.3% and 70% of the mothers with a single episode, 2 and 3 episodes respectively. In affected breasts, an episode of severe SCM resulted in steep upregulation of the four cytokines considered (IL8, IP10, RANTES and IL6) compared to: before and after the episode; contralateral unaffected breasts; and SCM negative control mothers. Recurrent severe SCM significantly increased the odds of shedding cell-free HIV-1 in breast milk (OR: 5.2; 95%CI: 1.7 – 15.6) whereas single episode of severe SCM did not (OR: 1.8; 95%CI: 0.8 – 4.2). A Na^+^/K^+^ ratio > 1 indicative of severe SCM is an excellent indicator of breast inflammation characterized by a steep, localized and temporal upregulation of several pro-inflammatory cytokines that favor HIV-1 shedding in mature breast milk and may facilitate postnatal HIV-1 transmission through breastfeeding.

## Introduction

The concept of subclinical mastitis (SCM) in humans is still poorly understood (1, 2) despite having been described since the late 90s (3, 4). Some authors describe SCM as opening of the tight junctions in the mucosal epithelial layer of the mammary gland resulting in increased membrane permeability (5–7). Others describe it as a local inflammation (2, 3, 8–13) or an infection (14, 15) or both infection and inflammation (16, 17) of the breast without clinical symptoms of mastitis.

SCM may be caused by several factors. These include milk stasis arising from inefficient milk removal (4, 18). Human breast milk ducts are branching tubular structures, easily compressed and without sinuses (19) suggesting that they are not meant for storage. Therefore, reduced milk removal results in distended mammary acini which ultimately leads to opening of tight junctions (20, 21). Milk stasis may be followed by an infection. Studies have reported increased concentration of innate immune response to bacterial exposure and bacteria lipopolysaccharide (LPS) in SCM affected breast milk samples suggesting a low-grade bacteria involvement (14). However, microbial culture fails to find putative organisms in the majority of SCM positive breast milk samples (16) and many women with potentially pathogenic bacteria in breast milk do not show any symptoms of mastitis (22).

Different diagnostic criteria for SCM are in use. Currently, the most widely accepted marker of SCM seems to be the breast milk sodium/potassium (Na^+^/K^+^) ratio. Na^+^/K^+^ ratio reflects increased permeability of the mucosal epithelial barrier allowing influx of interstitial Na^+^ into the mammary gland and subsequently into breast milk (8, 23, 24). Different Na^+^/K^+^ ratio thresholds are used to determine SCM. Some authors use the cut-off point of 1, above which they consider it to be SCM (4, 14, 25–27) while others use 0.6 as cut-off (3, 11, 15, 28–30). For those using 0.6 cut off, Na^+^/K^+^ ratio is further classified into moderate (above 0.6 to 1) and high (above 1) membrane permeability. Other markers previously used to determine SCM include raised sodium concentration (6, 8, 27) and increased breast milk leukocyte count (16, 27).

SCM is common among breastfeeding women in Zambia (25) and in sub-Saharan Africa (26, 28). In this region which is at the epicenter of the HIV-1 epidemic, several observational studies have shown that SCM is an important risk factor for mother-to-child transmission of HIV-1 (MTCT) through breast milk (15, 28, 30–32). This increased risk of postnatal HIV-1 transmission is attributed partly to increased cell-associated (CAV) and cell-free (CFV) HIV-1 shedding in breast milk observed in SCM (32, 33). In principle, in mothers on antiretroviral therapy (ART) this effect may be attenuated. The extent of this attenuation still needs to be assessed. However, breastfeeding mothers who seroconvert during the breastfeeding period and are unaware of their status (34) or those that have defaulted on their ART, SCM will exacerbate HIV-1 shedding in breast milk thereby increasing exposure to HIV-1 and HIV acquisition by breastfeeding infants. Other effects of SCM include alteration of breast milk’s immune factors (8, 14, 35) and trace elements composition (11) which may affect infants’ early development and growth (36, 37).

Most studies looking at SCM are not prospective in nature and therefore unable to follow the development of SCM over time and longitudinal effects of SCM on the mammary gland environment. Another common methodological weakness is analyzing only one sample from one breast (8, 38), thereby increasing the potential of underestimating SCM occurrence. In Zambia (25) and in the sub region (28), SCM has been well described based on studies of both breasts up to week 16 postpartum but its occurrence beyond 16 weeks remains relatively unexplored despite the fact that most of the mother to child HIV transmission (MTCT) currently observed occur late in the breastfeeding period (39, 40). Considering that SCM is an important risk factor of postnatal transmission of HIV (28, 30), there is need to clarify SCM beyond 16 weeks postpartum to complete SCM picture throughout the breastfeeding period. We conducted a longitudinal study to determine the prevalence of mothers with recurrent SCM and its association with the risk of HIV transmission. We also determined longitudinal effects of SCM on 4 pro-inflammatory cytokines, namely; interleukin-6 (IL6), interleukin 8-(IL8), interferon gamma-induced protein-10 (IP-10) and regulated upon activation, normal T-cell expressed and secreted (RANTES). Finally, we attempted to characterize these mothers for an early identification during the course of the breastfeeding period.

## Methods

### Study Design and Setting

This was a retrospective longitudinal sub-study nested in the ANRS12174 randomized trial (NCT00640263) that followed 1273 HIV-1 exposed infants for up to 50 weeks in Zambia, Uganda, South Africa and Burkina Faso from November, 2009 to May, 2012. Trial protocol (41) and main findings (42) have been published. None of the participating mothers received any antiretroviral drugs (ARVs) during breastfeeding but their newborns received infant antiretroviral prophylaxis (AZT and 3TC before randomization on day 7 of life and either 3TC or lopinavir/ritonavir (Kaletra) after randomization) for PMTCT. The study population for this sub-study was the 563 mother-infant pairs enrolled in the Zambian trial site using a large biobank of breast milk samples collected during the trial. Inclusion criteria were mothers who: (i) were randomized into the ANRS12174 trial in Zambia; (ii) returned for postpartum follow-up visits at week 14, 26 and 38; (iii) were still breastfeeding at week 38; and (iv) had breast milk samples from both breasts achieved at all these follow-up visits during the trial.

### Breast Milk Sample Collection and Processing

To collect breast milk, mothers were asked to express breast milk manually, from each breast, in separate sterile 50 ml polypropylene conical centrifuge tubes after breastfeeding. Collected breast milk samples were immediately transferred to the trial’s research laboratory within the University Teaching Hospital. Up on reception, breast milk samples were centrifuged at 1,200 g for 15 minutes at 4°C to separate the acellular aqueous fraction (lipid layer and lactoserum) and breast milk cells (BMCs). Both the acellular fraction and the dry BMC pellets were aliquoted and stored at -80°C.

### Sodium and Potassium Measurement

To measure Na^+^ and K^+^, the frozen acellular breast milk fraction was allowed to equilibrate to room temperature and centrifuged at 1,200 g for 15 minutes to separate lactoserum and the lipid layer. Clear lactoserum was diluted in 1:101 ratio with deionized water before testing. Na^+^ and K^+^ were measured in diluted lactoserum using a PFP 7 flame photometer (Jenway, Staffordshire, United Kingdom) according to manufacturer’s recommendations. A standard curve was built at the beginning of each batch of measurements using commercially available standard solutions (Jenway, Staffordshire, United Kingdom). Na^+^/K^+^ ratio was then calculated as a measure of SCM.

### HIV-1 and Cytokine Measurement

Both HIV-1 RNA and DNA were measured in breast milk from both breasts as previously described (33).

Four pro-inflammatory cytokines, namely IL6, IL8, IP10 and RANTES were measured using colorimetric sandwich enzyme-linked immunosorbent assay (ELISA) according to manufacturer’s recommendations (PeproTech, Stockholm, Sweden).

### Data Collection and Management

Na^+^, K^+^ and cytokine assay results were entered in an MS Excel worksheet and individually crosschecked for errors. The resulting clean worksheet was imported into SPSS version 25 (IBM Corporation, New York, USA) for analysis. Concurrent blood test results and sociodemographic data were extracted from the trial database and merged with the dataset of breast milk results using participants’ individual identifiers and study visit as matching variables. The resulting database was checked for errors and prepared for analysis.

### Statistical Analysis

In this study, samples with Na^+^/K^+^ ratio less than or equal to 0.6 (Na^+^/K^+^ ≤ 0.6) were classified as negative while those with Na^+^/K^+^ ratio greater than 0.6 to 1 (0.6 < Na^+^/K^+^ ≤ 1) were classified as mild SCM and those with Na^+^/K^+^ ratio greater than 1 (Na^+^/K^+^ > 1) classified as severe SCM, as previously described (13, 34). Descriptive statistics were used to summarize baseline characteristics of study participants. Percentages were reported for categorical variables. Either means with 95% confidence interval (CI) or median with interquartile range (IQR) were reported for continuous variables depending on whether the variables were normally distributed or not. Incidence of at least one episode of any SCM and severe SCM were determined using Kaplan-Meier survival model with comparison of factor levels done using the log Rank (Mantel–Cox) statistical test.

Based on the Na^+^/K^+^ ratio, participating mothers were divided in three groups; namely: SCM negative at all visits (SCM negative); mild SCM at least at one visit but no severe SCM at any visit (mild SCM); and severe SCM at least at one visit (severe SCM). From each group we randomly selected participants to include in the retrospective cohort study. We then determined normal ranges for cytokine concentration in mothers whose samples tested negative for SCM throughout the follow up period as 2.5 and 97.5 percentiles after removal of outliers (mean ± 3SD). We determined the proportion of samples with high cytokines in the different groups of SCM. Generalized linear mixed models were used directly or after log_10_ transformation to compare continuous variables between groups depending on whether the data was normally distributed or not. Fixed degrees of freedom were used with robust covariance and mean estimate tests were adjusted using sequential Bonferroni procedure with a significance level of 0.05. In a sensitivity analysis we compared the results within-breast and between breasts.

Multinomial logistic regression model was used to determine the effect of recurrent severe SCM on HIV-1 shedding in breast milk. All variables associated with the dependent model (p < 0.200) were included in the model (method: entre). CFV, CAV and SCM status were determined based on both breasts as previously described (33). We used Statistical package for social science (SPSS) version 25 (IBM Corporation, Armonk, New Yolk to perform statistical analysis.

### Ethical Considerations

All participants, at inclusion, had provided informed consent for sample storage and use in future research. Authority to conduct this sub-study was obtained from the National Health Research authority (NHRA) in Zambia and the scientific committee of the ANRS12174 trial. Ethical approval was obtained from ERES CONVERGE Institution Review Board in Zambia (IRB No. 00005948, FWA No. 00011697), and the Regional Committees for Medical Research Ethics of Norway (REK Nord no 2008 02523-2).

## Results

### Participants’ Characteristics

**Figure 1:**
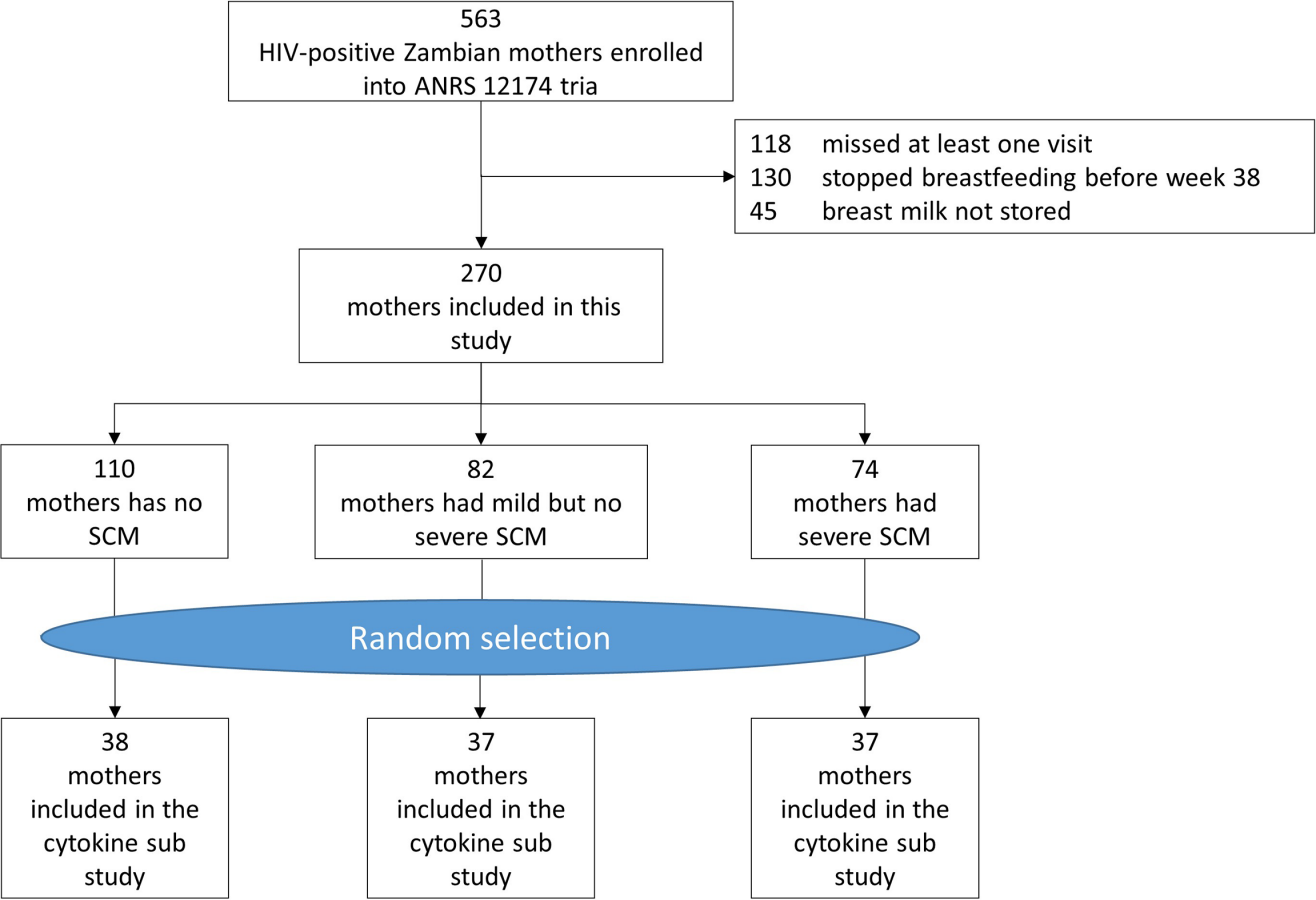
Participants flow chart.

270 mothers met inclusion criteria and were all included in this sub-study (**Figure 1**). Mothers’ characteristics are summarized in **Table 1**. Participants were between 18 and 44 years old with their 1st to 8th pregnancy. The majority of participating mothers were married (89.3%), unemployed (83.0%) prior to the current pregnancy and 69.2% had at least finished primary education (7 years of schooling). Most of the mothers initiated breastfeeding within 24 hours (99.6%).

**Table 1:** Participants’ characteristics.

Characteristics	Frequency	Percentage
**Demographic**		
Age		
< 20	22	8.1
20 - 29	167	61.9
30 - 39	75	27.8
≥ 40	6	2.2
Parity		
Primiparous	48	17.8
Multiparous	222	82.2
**Socioeconomic**		
Marital status		
Married/cohabiting	241	89.3
Single	19	7.0
Divorced	5	1.9
Widowed	5	1.9
Education level		
No school	25	9.3
Primary	58	21.5
End of primary	56	20.7
Secondary	91	33.7
End of secondary and more	40	14.8
Occupation		
Not working	224	83.0
Working	45	16.7
Studying	1	0.4
Source of water		
Piped water	229	84.8
Bore hole	39	14.4
Others	2	0.7
Has Electricity		
Yes	160	59.3
No	110	40.7
**Breastfeeding practice**		
Initiated breastfeeding within 24 hours		
Yes	269	99.6
No	1	0.4
Exclusive breastfeeding up to 6 months		
Yes	270	100.0
No	0	0.0
Ever had breast health problems		
Yes	7	2.6
No	263	97.4
Received antiretroviral drugs during pregnancy (PMTCT)		
Yes	270	100.0
No	0	0.0

### Cohort Description Over Time

270 breastfeeding mothers were followed for a median time of 38 weeks (IQR: 38 – 38; range: 36 – 41). They practiced exclusive breastfeeding (100%) up to six months. Breast health problems were rare with only 7 (2.6%) mothers ever reporting. Only one of these (week 14 case) required antibiotic treatment for 5 days. The proportion of mothers with unsuppressed viral load (≥1000 copies/ml) increased over the duration of the study, from 50.2% at day 7 to 87.0% at week 38 postpartum. CD4 cell count did not change significantly over time.

Of the 270 mothers included in the study, 1610 breast milk samples were available for Na^+^ and K^+^ analysis (**Table 2**). The concentration of K^+^ in lactoserum significantly reduced between week 14 and week 26 (p < 0.001) but remained stable between week 26 and week 38 while that of Na^+^ reduced significantly between week 14 and week 26 (p = 0.004) and tended to increase between week 26 and week 38 (p = 0.072). On the other hand, lactoserum Na^+^/K^+^ ratio remained stable throughout the follow up period.

**Table 2:** Time effect on systemic HIV and indicators of subclinical mastitis (SCM).

	CD4 count in cells/µl	Log_10_ Plasma HIV-1 viral load	Potassium concentration in mmol/l	Sodium concentration in mmol/l	Sodium/Potassium ratio
	Mean (95%CI)	Mean (95%CI)	Mean* (95%CI)	Mean* (95%CI)	Mean* (95%CI)
During pregnancy	598 (572 - 624)	–	–	–	–
Day 7	–	3.2 (3.0 - 3.3)	–	–	–
Week 14	673 (644 - 702)	4.2 (4.1 - 4.3)	14.3 (14.0 - 14.6)	7.4 (6.9 - 8.0)	0.5 (0.5 - 0.6)
Week 26	–	–	13.9 (13.6 - 14.1)	6.7 (6.3 - 7.2)	0.5 (0.4 - 0.5)
Week 38	650 (621 - 679)	4.4 (4.2 - 4.5)	13.9 (13.6 - 14.1)	7.2 (6.8 - 7.7)	0.5 (0.5 - 0.6)

*Geometric mean.

### Incidence of SCM Between Week 14 and Week 38 Postpartum

The prevalence of any SCM (Na^+^/K^+^ ratio > 0.6) at week 14, week 26 and week 38 was 35.6%, 36.8% and 41.9%, respectively, while that of severe SCM (Na^+^/K^+^ ratio > 1) was 16.5%, 15.2% and 17.4%, respectively. Bilateral severe SCM accounted for 4.5%, 7.3% and 21.3% of those with severe SCM at week 14, week 26 and week 38, respectively.

Overall cumulative incidence of any SCM between week 14 and week 38 was 58.6% (95%CI: 52.7 – 64.5) while that of severe SCM was 27.8% (95%CI: 22.5 – 33.1). The incidence of severe SCM was significantly higher (p = 0.002) in those who had mild SCM at week 14 (25.5%) compared to those who had no SCM at the same time point (9.4%). Of the 74 mothers that had severe SCM, 36 (48.6%) had a single episode, 18 (24.3%) had two episodes while the remaining 20 (27.0%) had three episodes. Both breasts were affected over time, but not necessarily at the same time, in 11.1%, 33.3% and 70.0% of those who had 1, 2 and 3 episodes of severe SCM.

### Association Between SCM and Pro-Inflammatory Cytokines (IL8, IP10, RANTES and IL6)

**Figure 2:**
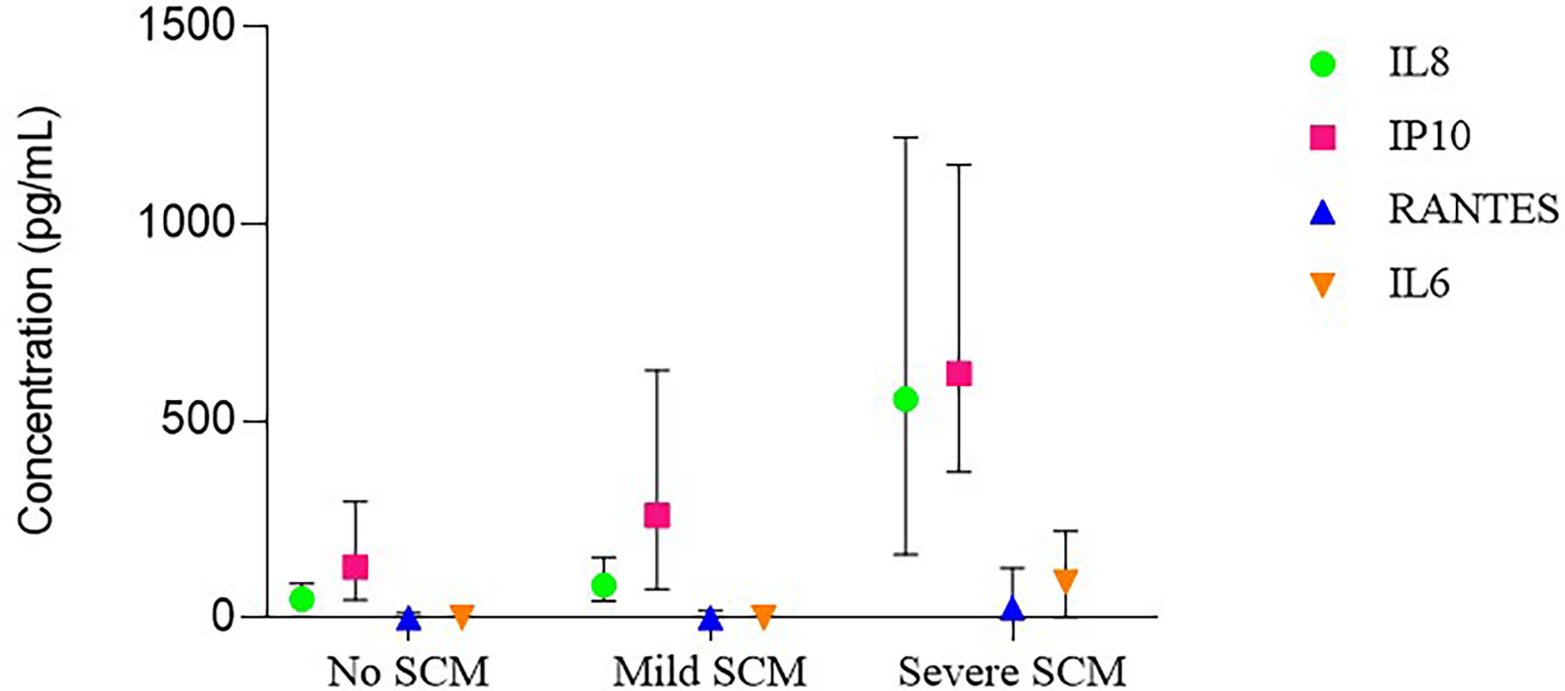
Cytokine concentrations in milk lactoserum according to subclinical matitis (SCM) status.

To study the effects of SCM on breast milk cytokines over time, 672 breast milk samples were retrieved, accounting for 38 SCM negative mothers, 37 mild SCM mothers and 37 severe SCM mothers (**Figure 2** and **Supplementary Table 1**).

The normal range for IL8 concentration in lactoserum was from 0 to 286 pg/ml. High IL8 concentration (> 286 pg/ml) was observed in 3.3%, 7.0% and 62.3% of samples with no, mild and severe SCM, respectively. During an episode of either mild or severe SCM, IL8 concentration increased about 2- and 9-fold, respectively, compared to SCM negative mothers. After any type of SCM, IL8 concentration returned to pre SCM levels.

The normal range for IP10 concentration in lactoserum was from 6 to 1010 pg/ml. High IP10 concentration (> 1010 pg/ml) was observed in 3.8%, 12.2% and 31.2% of samples with no, mild and severe SCM, respectively. There was a tendency for IP10 concentration to increase during mild SCM but the increase was not statistically significant. However, during an episode of severe SCM, the IP10 concentration increased about 4-fold compared to SCM negative mothers. After any type of SCM, the concentration of IP10 retuned to pre SCM levels.

RANTES was more likely to be detected in samples with severe SCM (70.1%) compared to those with mild SCM (44.3%) and no SCM (38.0%). The normal range for RANTES concentration in lactoserum was from 0 to 39 pg/ml. High RANTES concentrations (> 39 pg/ml) was observed in 4.4%, 11.3% and 39.0% of samples with no, mild and severe SCM, respectively. RANTES concentration did not change significantly during mild SCM. However, during an episode of severe SCM, RANTES concentration increased about 4-fold compared to SCM negative mothers. After severe SCM, the concentration of RANTES retuned to pre SCM levels.

IL6 was more likely to be detected in samples with severe SCM (57.1%) compared to those with mild SCM (20.9%) and no SCM (22.7%). The normal range for IL6 concentration in lactoserum was from 0 to 74 pg/ml. High IL6 concentration (> 74 pg/ml) was observed in 3.8%, 7.0% and 35.1% of samples with no, mild and severe SCM, respectively. IL6 concentration did not change significantly during mild SCM. However, during an episode of severe SCM, the concentration of IL6 increased about 5-fold compared to SCM negative mothers. After severe SCM, IL6 concentration retuned to pre SCM levels.

**Figure 3:**
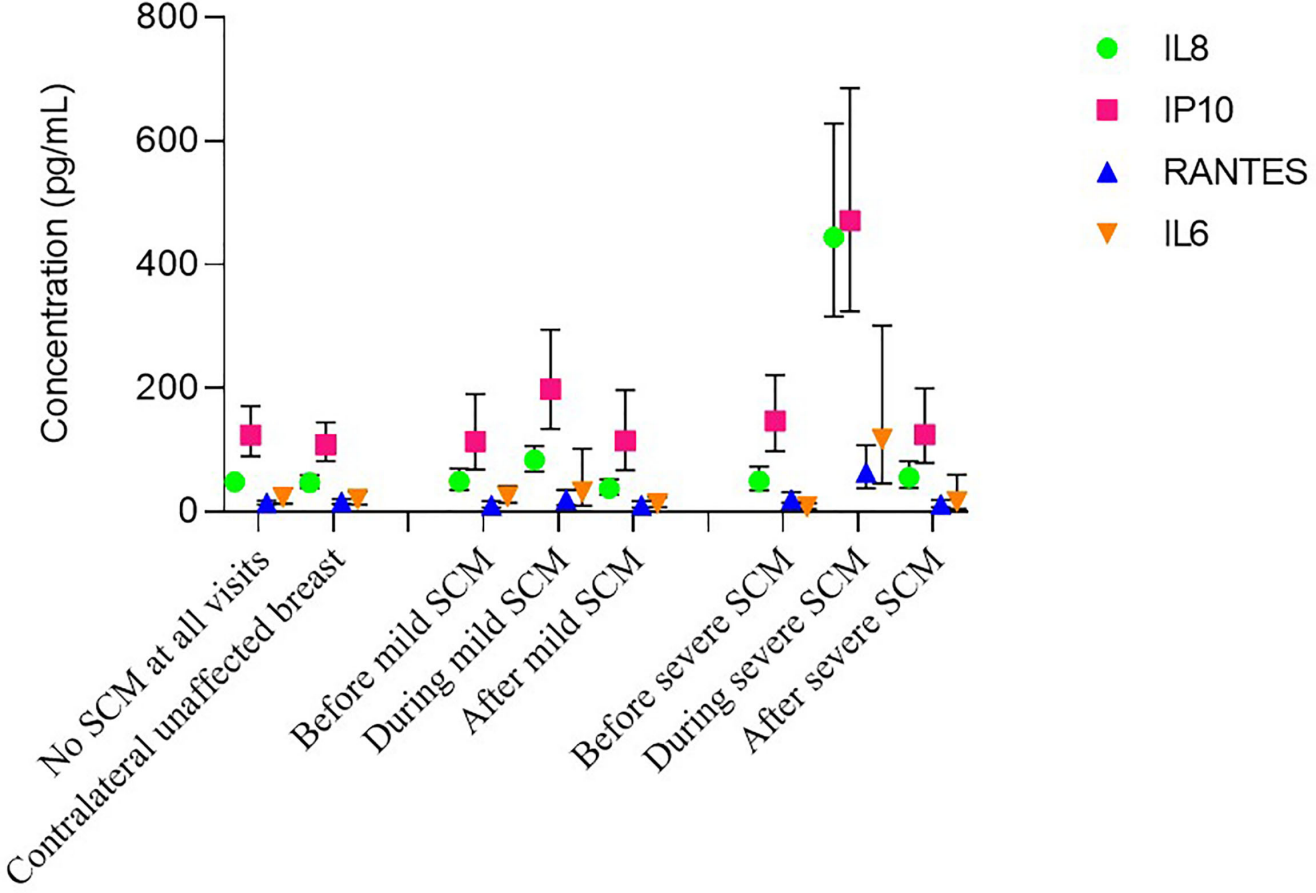
Dynamics of cytokine concentrations during subclinical mastitis (SCM) episode.

For the four cytokines, their concentration among SCM negative mothers (controls) was similar to that of unaffected contralateral breasts in unilateral SCM. In affected breasts, cytokine concentrations before either mild or severe SCM was similar to that of SCM negative mothers too (**Figure 3**).

### Recurrence of SCM and the Risk of HIV Transmission

Severe SCM increased CFV HIV-1 shedding in breast milk 7 folds from a median of 41 copies/mL (IQR: 0 – 179) mild SCM to a median of 309 copies/mL (IQR: 18 – 1940) in severe SCM. The median CFV-1 HIV shedding in SCM negative mothers was 0 copies/mL (IQR: 0 – 71). Similar trends were observed for CAV HIV-1 shedding and total breast milk cell count (**Table 3**). In bivariate analysis, recurrent severe SCM was not associated with any of the sociodemographic, anthropometric and breastfeeding practice maternal characteristics when compared to mothers with no severe SCM. These variables were not statistically different between mothers with single or multiple severe SCM episodes (**Supplementary Table 2**). In multivariable analysis, recurrent severe SCM was significantly associated with increased shedding CFV in breast milk at week 38 while single episodes of severe SCM did not (**Table 4**). Similar analysis indicated that both single episode and recurrent severe SCM significantly increase CAV shedding in breast milk (**Table 5**). There was indication that use of hormonal contraceptives was associated with recurrent severe SCM.

**Table 3:** Cell free HIV-1 virus (CFV), cell-associated HIV-1 virus (CAV) and Breast Milk Cells (BMC) concentration in breast milk during no, mild and severe subclinical mastitis (SCM).

Subclinical mastitis	CFV HIV-1 viral load in copies/ml	CAV HIV-1 viral load in copies/10^6^ cells	Plasma HIV-1 viral load in copies/ml
	Median (IQR)	Median (IQR)	Median (IQR)
No SCM	0 (0 - 71)	0 (0 - 12)	25,704 (4,940 – 97,500)
Mild SCM	41 (0 – 179)	0 (0 – 40)	23,331 (3,595 – 107,640)
Severe SCM	309 (18 - 1905)	20 (0 – 131)	16,263 (2,788 – 140,040)

**Table 4:** Effect of recurrent severe sub-clinical mastitis (SCM) on cell-free viral (CFV) shedding in breast milk.

Recurrent Severe SCM*	Factors	Crude OR			Adjusted OR		
		OR	95%CI	*p* value	OR	95%CI	*p* value
Single episode	Exclusive breastfeeding up to week 26						
	Yes	1			1		
	No	0.3	0.1 - 1.4	0.136	0.2	0.2 - 1.3	0.175
	Cell-free HIV-1 shedding at week 38						
	Not detected	1			1		
	Detected	1.9	0.9 - 4.2	0.126	1.8	0.8 - 4.2	0.168
	Hemoglobin concentration at week 38						
	≥12 g/dl	1					
	<12 g/dl	1.6	0.8 - 3.5	0.216	1.7	0.8 - 3.6	0.188
	Use of oral hormonal contraceptives at week 38						
	No	1					
	Yes	1.6	0.8 - 3.3	0.202	1.7	0.8 - 3.6	0.188
Multiple episodes	Exclusive breastfeeding up to week 26						
	Yes	1			1		
	No	0.5	0.1 - 1.7	0.278	0.4	0.1 - 1.8	0.223
	Cell-free HIV-1 shedding at week 38						
	Not detected	1			1		
	Detected	**4.2**	**1.6 - 11.2**	**0.005**	**5.2**	**1.7 - 15.6**	**0.003**
	Hemoglobin concentration at week 38						
	≥12 g/dl	1			1		
	<12 g/dl	1.7	0.8 - 3.5	0.177	1.2	0.5 - 2.7	0.669
	Use of oral hormonal contraceptives at week 38						
	No	1			1		
	Yes	1.6	0.8 - 3.2	0.187	2.0	0.9 - 4.4	0.072

*Reference category is no severe SCM.

Bold values are statistically significant.

**Table 5:** Effect of recurrent severe subclinical mastitis (SCM) on cell-associated viral (CAV) shedding in breast milk.

Recurrent Severe SCM*	Factors	Crude OR			Adjusted OR		
		OR	95%CI	*p* value	OR	95%CI	*p* value
Single episode	Exclusive breastfeeding up to week 26						
	Yes	1			1		
	No	0.3	0.1 - 1.4	0.136	0.2	0.3 - 1.5	0.113
	Cell-associated HIV-1 shedding at week 38						
	Not detected	1			1		
	Detected	**3.2**	**1.3 - 7.6**	**0.010**	**3.2**	**1.3 - 8.0**	**0.011**
	Hemoglobin concentration at week 38						
	≥12 g/dl	1					
	<12 g/dl	1.6	0.8 - 3.5	0.216	1.6	0.7 - 3.6	0.243
	Use of oral hormonal contraceptives at week 38						
	No	1					
	Yes	1.6	0.8 - 3.3	0.202	1.7	0.8 - 3.8	0.182
Multiple episodes	Exclusive breastfeeding up to week 26						
	Yes	1			1		
	No	0.5	0.1 - 1.7	0.278	0.4	0.1 - 1.8	0.243
	Cell-associated HIV-1 shedding at week 38						
	Not detected	1			1		
	Detected	**4.2**	**1.7 - 10.6**	**0.002**	**5.3**	**1.9 - 14.4**	**0.001**
	Hemoglobin concentration at week 38						
	≥12 g/dl	1			1		
	<12 g/dl	1.7	0.8 - 3.5	0.177	1.4	0.6 - 3.2	0.440
	Use of oral hormonal contraceptives at week 38						
	No	1			1		
	Yes	1.6	0.8 - 3.2	0.187	2.2	1.0 - 4.7	0.055

*Reference category is no severe SCM.

Bold values are statistically significant.

## Discussion

Our study demonstrated that: first, SCM is common among breastfeeding HIV-1 positive mothers later during the breastfeeding period (beyond 14 weeks). Second, a Na^+^/K^+^ ratio of 1 is more relevant than 0.6 in as far as mammary gland inflammation is concerned. Thirdly, severe SCM corresponding to a Na^+^/K^+^ ratio > 1 is common, recurrent and accompanied by steep upregulation of pro-inflammatory cytokines in the mammary gland. Lastly, recurrent severe SCM is associated with increased HIV-1 shedding in breast milk and may increase the risk of HIV transmission through breastfeeding.

These results compare well with earlier similar studies. For instance, we report a 16.5% point prevalence of severe SCM at week 14 which is in line with similar studies in the same location (25) and in the sub-region (3, 28, 31). Our study complements these earlier studies which covered the early breastfeeding period (day 3 to week 16 postpartum) to cover the later breastfeeding period (week 14 to week 38). On the other hand, earlier studies in the same population and in the sub region that used either sodium concentration (43) or milk leucocyte count (16) to determine SCM generally reported lower proportions of mothers with SCM.

The establishment of lactogenesis II and copious production of breast milk is heralded by closure of tight junctions essentially separating interstitial fluid and breast milk (44). This reduces sharply the influx of interstitial Na+ into breast milk which explains the drastic reduction in Na^+^/K^+^ ratio and the severe SCM observed in the first week (day 3 to day 7) postpartum followed by a gradual reduction up to week 16 (25). Our study goes on to show that both the prevalence of severe SCM and the Na^+^/K^+^ ratio remain relatively stable beyond 14 weeks thereby suggesting that severe SCM before and after 14 weeks could be driven by different factors. Indeed opening of tight junctions at the onset of lactogenesis II may be a normal physiological process to allow transfer of material from mother to the newborn (21, 45). In our study, Na^+^ concentration fluctuated significantly over time while K^+^ concentration reduced significantly over time until week 26 and remained stable thereafter. Na^+^/K^+^ ratio remained stable throughout the follow-up period thereby confirming the superiority of Na^+^/K^+^ ratio over Na^+^ concentration as an indicator of SCM.

Our results indicate that between week 14 and week 38, about 58.6% of breastfeeding HIV-1 infected mothers had at least 1 episode of any SCM while 27.8% had at least 1 episode of severe SCM. Like in previous studies (5, 25, 26), severe SCM episodes were mainly unilateral. However, about half (51.4%) of the mothers who had severe SCM had multiple (2 or more) episodes and in a majority (52.6%) of these mothers with recurrent severe SCM both breasts were affected over time but rarely at the same time, thereby confirming that these were different episodes.

Such a high incidence for SCM was surprising as our participants received infant feeding counselling at each visit during the trial, which has been previously associated with lower incidence of SCM (46). Therefore, factors other than breastfeeding practices and milk removal may be contributing to SCM. Indeed stress (47), depression (7), micronutrient deficiency (35), vitamin E supplementation (13), general maternal health status (25), bacterial infection in the breast (14, 48) viral shedding in breast milk (49, 50), infant ART prophylaxis (51), and systemic inflammation (3) have all been associated with SCM. Despite high incidence of severe SCM, however, breast health problems were very rare with only 1 mother reporting breast health problems (at week 14) between week 14 and week 38 implying that most of the severe SCM cases resolved spontaneously without presenting any symptoms and consequently without affecting breastfeeding.

Like in earlier studies (5, 15, 30), we report association between HIV-1 shedding in breastmilk and severe SCM with additional report that recurrent severe SCM worsens both CFV and CAV shedding in breast milk thereby increasing the risk of breast milk transmission of HIV-1. Whether this increase is a consequence or a cause of severe SCM still need to be determined. Although we were unable to predict mothers who will develop recurrent severe SCM, our results indicate that mothers who report mild SCM and severe SCM early in the breastfeeding period (Week 14) are more likely to develop severe SCM and recurrent severe SCM, respectively, later on in the breastfeeding period compared to those who are SCM negative at week 14. In the case of CAV, HIV cell reservoirs have been described (52) and include mammary epithelial cells, macrophages, lymphocytes and stem cells. In the mammary epithelial cells reservoir, HIV is thought to be unintegrated but it forms an inducible and functional reservoir capable of initiating HIV replication in susceptible cells in mothers not on ART (53). To prevent MTCT associated with mastitis, WHO recommends that HIV-1 infected breastfeeding women with mastitis stop breastfeeding on the affected breast and breastfeed on the contralateral unaffected breast if unilateral until symptoms resolve (4). However, this advice is not useful for recurrent severe SCM in late breastfeeding which has no overt symptoms and involves both breast most over time but potentially has similar effects as clinical mastitis on HIV-1 transmissions through breastfeeding (15, 28, 30, 31).

Severe SCM as defined by Na^+^/K^+^ ratio is a measure of mammary epithelial layer permeability (21). High permeability (severe SCM) facilitates microbial translocation through epithelial barriers (50) and influx of systemic materials into the mammary gland. This allows antigen processing by resident immune system in the mammary gland, which triggers the classic inflammation observed in severe SCM. Here we report the longitudinal dynamics of this association. Although several studies have reported an association between severe SCM and pro-inflammatory cytokines in breast milk (8, 9, 14, 38) these reports were mostly based on between-subjects analysis at a single time point and the sample sizes were relatively small. In addition to between-subjects analysis, we also did within-subject analysis comparing unaffected breasts to affected breasts and within-breast analysis comparing affected breasts before during and after an episode of SCM thereby eliminating confounding. This coupled with the large number of samples analysed and robust analysis procedures make us believe that there is a strong relationship between severe SCM and increased cytokine levels in breast milk. The consistency of between subjects, between breasts and within breast results also strengthens validity of our results. Whether this increase in cytokine concentrations is the cause or consequence of SCM remains to be elucidated.

Our results are consistent with those of other studies (8, 9, 14, 38) with an additional proof that the longitudinal profile of inflammation markers (IL8, IP10, RANTES and IL6) closely follow that of Na^+^/K^+^ ratio. Our results also demonstrate that inflammation associated with severe SCM is localized within the affected breast and temporal only occurring during an active episode of severe SCM. However, because not all samples that had severe SCM and some samples with no SCM had high cytokine levels, there may be other factors contributing to mammary gland inflammation. Indeed, it has been reported that HIV-1 glycoprotein 120 (gp120) triggers production of pro-inflammatory cytokines (54). In line with this notion, ART has also been associated with suppression of circulatory inflammatory markers (55, 56). Other factors that have been shown to impact breast milk inflammation makers are systemic maternal inflammation (3), origin (57), active infection in the infant (58) and allergies (59).

Of the 4 cytokines considered, IP10 and IL8 were the most common, detectable in almost all samples and in similar quantities. RANTES were detectable in about half of the samples while IL6 was the least common detectable only in about a quarter of samples. Both RANTES and IL6 occurred in much lower quantities compared to IP10 and IL8. A recent study (14) detected IL6 and RANTES in all samples tested and generally reported higher absolute concentrations compared to our results. However, like in our study, RANTES and IL6 occurred in lower quantities compared to IP10 and IL8. Based on the median ratio in severe SCM samples (similar definition as SCM in the other study) results reported by Tuaillon et al., 2016 were about 8-fold higher for IL8, RANTES and IL6, and about 6-fold high for IP10. Despite these differences, quantitative trends were similar (14). For instance, severe SCM over no severe SCM median ratio for IL8 was 9.6 in Tuaillon et al., 2016 compared to 10.1 in our study. For IP10 it was 4.2 versus 4.2, for RANTES it was 3.7 versus 3.2 and for IL6 the increase was 7.1 versus 5.5. The differences in absolute value were mainly due to different methods used. Tuaillon et al., 2016 used Luminex multiplex bead assay, which is known to produce higher absolute quantities compared to classical ELISA used in this study while maintaining quantitative trends (60, 61).

The fact that all the 4 cytokines considered in this study were increased during severe SCM is an indication that there is a full-fledged inflammation going on during severe SCM. Consistent with this notion, studies showed that a wide spectrum of inflammatory markers are raised during severe SCM (9, 14). Some of the upregulated markers (IL6, TNFα, IFNγ and IL1β) have been associated with disruption of mucosal epithelial barrier function resulting in increased permeability (62–69). TNFα, IL1β and IL6 also promote HIV-1 replication and persistence of low range viremia in patients on successful ART (70, 71). Other cytokines raised during inflammation associated with severe SCM (IL8, IP10, RANTES, TNFα) have chemotactic functions (72–75) recruiting HIV-1 target cells into the site of inflammation. RANTES has also been associated with increased risk of breastfeeding transmission of HIV-1 (76).

Taken together therefore, we propose that inflammation associated with severe SCM exacerbates membrane permeability and promotes local replication of HIV-1 in the mammary gland. Increased membrane permeability allows increased translocation of cell-free HIV-1 particles into the mammary gland thereby explaining increased cell-free HIV-1 shedding in breast milk. On the other hand, persistence of low range viremia in the mammary gland maintains cell-associated HIV-1 reservoirs thereby explaining continued shedding of breast milk cell-associated HIV-1 (33, 77) and this could potentially account for periodic viral blips observed in breast milk of HIV-1 infected breastfeeding mothers on successful ART (72). Increased HIV-1 shedding coupled with a pro-inflammatory environment in infant’s gut would complement each other to increase the risk of HIV-1 transmission through breastfeeding either by cell to cell spread of HIV-1 (78) or by quickly initiating HIV-1 replication in the infant’s gut following easy translocation of HIV-1 particles through a permeable gut epithelial barrier to the gut mucosa lamina propria where recruited HIV-1 target cells home. Pro-inflammatory environment in the gut may also adversely affect maturity of the gut thereby putting these children at risk of other health problem later in life (14, 29, 36) even if they do not acquire the HIV-1 infection.

The limitations of our study include the long period between visits suggesting that we could have missed SCM episodes thus our estimates of incidence and number of episodes may be underreported. For the same reason we were unable to determine the duration of an SCM episode. Secondly our study only included breastfeeding HIV positive mothers, therefore our results may not be extrapolated to breastfeeding HIV negative mothers since HIV status has been associated with increased risk of SCM (25) and a dumped immune response during SCM (9). Finally, the low rate of HIV-1 transmission in this trial did not allow us to conclude on its association with recurrent severe SCM.

## Conclusion

A Na^+^/K^+^ ratio > 1 indicative of severe SCM is an excellent indicator of breast inflammation characterized by a steep, localized and temporal upregulation of several pro-inflammatory cytokines that favor HIV-1 shedding in mature breast milk which may facilitate postnatal HIV-1 transmission through breastfeeding.

## Data Availability Statement

The raw data supporting the conclusions of this article will be made available by the authors, without undue reservation.

## Ethics Statement

Ethical approval was obtained from ERES CONVERGE Institution Review Board in Zambia (IRB No. 00005948, FWA No. 00011697), and the Regional Committees for Medical Research Ethics of Norway (REK Nord no 2008 02523-2). The patients/participants provided their written informed consent to participate in this study.

## Author Contributions

All authors contributed substantially to the design, conduct, data analysis and results interpretation. DR drafted the paper. All authors reviewed and approved the final version of the manuscript.

## Funding

This work was supported by the French National Agency for Research on AIDS and Viral Hepatitis (ANRS#12274), European & Developing Countries Clinical Trials Partnership (#CT.2006.33020.004) and the Research Council of Norway (GlobVac grant # 183600). DR is the beneficiary of doctoral scholarship from the Norwegian government (Quota scheme).

## Conflict of Interest

The authors declare that the research was conducted in the absence of any commercial or financial relationships that could be construed as a potential conflict of interest.

## Publisher’s Note

All claims expressed in this article are solely those of the authors and do not necessarily represent those of their affiliated organizations, or those of the publisher, the editors and the reviewers. Any product that may be evaluated in this article, or claim that may be made by its manufacturer, is not guaranteed or endorsed by the publisher.
