# Supplementary material for: Recurrent Severe Subclinical Mastitis and the Risk of HIV Transmission Through Breastfeeding
Source: Front Immunol. 2022 Mar 4;13:822076. doi: 10.3389/fimmu.2022.822076 (PMC8931278; doi:10.3389/fimmu.2022.822076)
Supplement: Supplementary file 1 [file Table_1.docx]

**Supplementary Table 1: Effects of subclinical mastitis on pro-inflammatory cytokines**

|  | **IL8 concentration in pg/ml** | **IP10 concentration in pg/ml** | **RANTES concentration in pg/ml** | **IL6 concentration in pg/ml** |
| --- | --- | --- | --- | --- |
|  | **Mean* (95%CI)** | **Mean* (95%CI)** | **Mean* (95%CI)** | **Mean* (95%CI)** |
| No SCM at all visits | 48 (40-58) | 124 (90-171) | 14 (12-18) | 23 (13-38) |
|  |  |  |  |  |
| Before mild SCM | 49 (35-70) | 114 (68-190) | 10 (6-17) | 24 (14-42) |
| During mild SCM | 83 (65-106) | 198 (133-294) | 20 (11-35) | 32 (10-101) |
| After mild SCM | 37 (27-52) | 115 (67-197) | 10 (6-17) | 13 (7-24) |
|  |  |  |  |  |
| Before severe SCM | 50 (34-73) | 14 (98-221) | 20 (14-32) | 8 (5-14) |
| During severe SCM | 445 (316-628) | 471 (324-686) | 64 (38-107) | 117 (46-301) |
| After severe SCM | 56 (38-82) | 125 (78-200) | 12 (7-19) | 16 (4-60) |
|  |  |  |  |  |
| Contralateral, unaffected breasts | 47 (38-59) | 109 (82-144) | 16 (12-21) | 19 (11-33) |
| *: Geometric mean |  |  |  |  |

**Supplementary Table 2: Bivariate analysis of the risk of severe SCM**

|  | No severe SCM (%) | Single episode of SCM (%) | Multiple episode of SCM (%) | p-value |
| --- | --- | --- | --- | --- |
| Age (year) |  |  |  | 0.47 |
| < 20 | 16 (73) | 4 (18) | 2 (9) |  |
| 20 - 29 | 115 (70) | 25 (15) | 25 (15) |  |
| 30 - 39 | 58 (80) | 6 (8) | 9 (12) |  |
| 40 - 49 | 3 (50) | 1 (17) | 2 (33) |  |
| Parity |  |  |  | 0.48 |
| Primiparous | 36 (75) | 4 (8) | 8 (17) |  |
| Multiparous | 156 (72) | 32 (15) | 30 (14) |  |
| BMI |  |  |  | 0.58 |
| <18.5 | 15(71 | 5 (24) | 1 (5) |  |
| 18.5 - 24.9 | 113 (72) | 21 (13) | 24 (15) |  |
| 25 - 29.9 | 46 (73) | 8 (13) | 9 (14) |  |
| ≥ 30 | 14 (74) | 1 (5) | 4 (21) |  |
| Education level |  |  |  | 0.27 |
| No school | 19 (76) | 1 (4) | 5 (20) |  |
| Primary | 33 (59) | 12 (21) | 11 (20) |  |
| End of primary | 43 (77) | 8 (14) | 5 (9) |  |
| Secondary | 66 (73) | 11 (12) | 13 (14) |  |
| End of Secondary | 31 (80) | 4 (10) | 4 (10) |  |
| BF within the first 24 hr |  |  |  | N.A. |
| No | 1 (100) | 0 (0) | 0 (0) |  |
| Yes | 191 (72) | 36 (14) | 38 (14) |  |
| HIV plasma VL at Day 7 |  |  |  | 0.34 |
| < 1000 cp/mL | 96 (73) | 13 (12) | 17 (15) |  |
| ≥ 1000 cp/mL | 89 (71) | 21 (15) | 17 (14) |  |
| HIV plasma VL at Month 9 |  |  |  | 0.84 |
| < 1000 | 23 (70) | 4 (12) | 6 (18) |  |
| ≥ 1000 | 157 (71) | 31 (14) | 32 (15) |  |
| EBF up to 6M |  |  |  | 0.18 |
| No | 29 (85) | 2 (6) | 3 (9) |  |
| Yes | 160 (71) | 34 (15) | 33 (15) |  |
| Any HIV shedding in BM at Month 9 |  |  |  | 0.011 |
| No | 47 (89) | 4 (8) | 2 (4) |  |
| Yes | 142 (69) | 30 (15) | 35 (15) |  |
| Baby Gender |  |  |  | 0.77 |
| Boy | 103 (73) | 17 (12) | 21 (15) |  |
| Girl | 89 (71) | 19 (15) | 18 (14) |  |
| HIV transmission |  |  |  | N.A. |
| No | 187 (72) | 36 (14) | 38 (15) |  |
| Yes | 5 (100) | 0 (0) | 0 (0) |  |

SCM : severe subclinical mastitis ; BMI: body mass index; BF: breastfeeding; PMTCT: program of mother-to-child transmission; VL: viral load; EBF exclusive breastfeeding; BM: breast milk; N.A. : not applicable.
